# Remarkable effect of alkalis on the chemoselective hydrogenation of functionalized nitroarenes over high-loading Pt/FeO_*x*_ catalysts†
†Electronic supplementary information (ESI) available: Experimental details, material characterization data, and catalytic measurement details. See DOI: 10.1039/c7sc00568g



**DOI:** 10.1039/c7sc00568g

**Published:** 2017-05-16

**Authors:** Haisheng Wei, Yujing Ren, Aiqin Wang, Xiaoyan Liu, Xin Liu, Leilei Zhang, Shu Miao, Lin Li, Jingyue Liu, Junhu Wang, Guofu Wang, Dangsheng Su, Tao Zhang

**Affiliations:** a State Key Laboratory of Catalysis , iChEM , Dalian Institute of Chemical Physics , Chinese Academy of Sciences , Dalian 116023 , China . Email: aqwang@dicp.ac.cn ; Email: taozhang@dicp.ac.cn; b University of Chinese Academy of Sciences , Beijing 100049 , China; c Department of Physics , Arizona State University , Tempe , Arizona 85287 , USA; d State Key Laboratory of Coal Conversion , Institute of Coal Chemistry , Chinese Academy of Sciences , Taiyuan 030001 , China

## Abstract

The chemoselective hydrogenation of substituted nitroarenes to form the corresponding functionalized anilines is an important type of reaction in fine chemistry, and the chemoselectivity is critically dependent on the rational design of the catalysts. This reaction has rarely been accomplished over high-loading Pt catalysts due to the formation of Pt crystals. Here, for the first time, we report that alkali metals (Li^+^, Na^+^, K^+^, *etc.*) can transform the non-selective high loading Pt/FeO_*x*_ catalyst to a highly chemoselective one. The best result was obtained over a 5% Na–2.16% Pt/FeO_*x*_ catalyst, which enhanced the chemoselectivity from 66.4% to 97.4% while the activity remained almost unchanged for the probe reaction of 3-nitrostyrene hydrogenation to 3-aminostyrene. Using aberration-corrected HAADF-STEM, *in situ* XAS, ^57^ and Fe Mössbauer and DRIFT spectroscopy, the active site of a Pt–O–Na–O–Fe-like species was proposed, which ensures that the Pt centers are isolated and positively charged for the preferential adsorption of the –NO_2_ group.

## Abstract 2

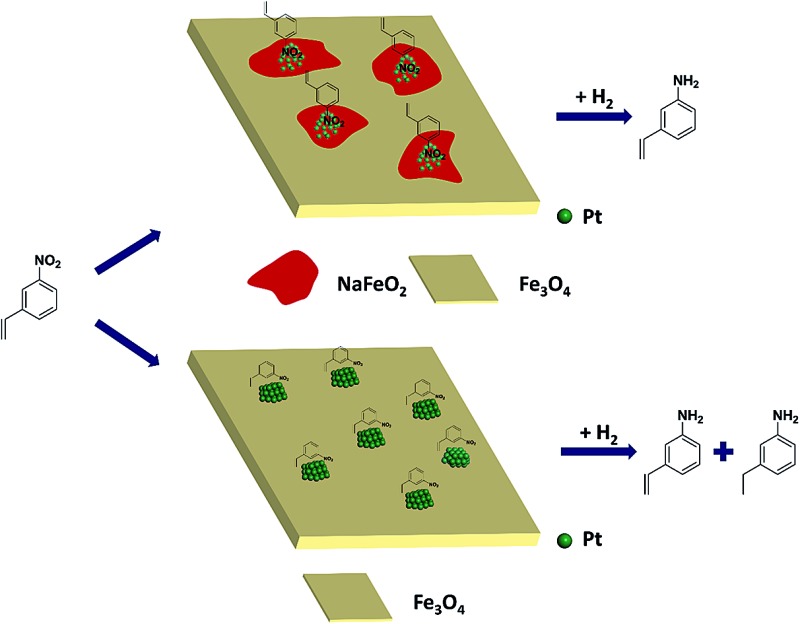
Alkali metals transform non-selective high-loading Pt/FeO_*x*_ to a highly chemoselective catalyst by helping to form isolated and electropositive Pt centers.

## Introduction

Chemoselective hydrogenation of substituted nitroarenes is a class of reaction which produces functionalized anilines, which are important intermediates for agrochemicals, pharmaceuticals and dyes.^1^ A big challenge for this reaction is the selective reduction of the nitro group when other reducible groups (*e.g.*, C=C, C=O, –X, *etc.*) are present because most transition metal catalysts cannot discriminate between different functional groups for selective hydrogenation. Therefore, great efforts have been made to seek highly chemoselective catalysts for the preferential hydrogenation of the nitro group in the presence of other reducible groups.^2^ Effective approaches reported to date include the introduction of a less active component (*e.g.*, Pt–Pb/CaCO_3_ catalyst),^2*a*^ partially covering the active metal surface with a reducible oxide support (*e.g.*, Pt/TiO_2_ catalyst),^2*b*^ as well as employing relatively inert metal (such as Au and Ag) catalysts.^3^ All of these strategies, however, enhance the chemoselectivity at the cost of activity, which is undesirable in practical applications. Therefore, it is keenly desired to develop highly chemoselective catalysts without compromising their activity.

Single-atom catalysts (SACs), which feature exclusive atomic dispersion of the active metals,^4^ have demonstrated distinctive selectivities in a variety of hydrogenation reactions without compromising their intrinsic activity.^5^ In particular, Pt_1_/FeO_*x*_ SAC showed a turnover frequency (TOF) of 1500 h^–1^ and a selectivity of close to 99% in the chemoselective hydrogenation of 3-nitrostyrene.^5*a*^ The outstanding performance of the Pt_1_/FeO_*x*_ SAC was attributed to the positively charged and isolated Pt center, which was supposed to be favorable to the preferential adsorption of the nitro group. In spite of their superior activity per metal atom, SACs usually have a pretty low metal loading (*e.g.*, <0.1 wt%), which is less attractive in practical applications due to the very small number of total active sites per weight of catalyst. Taking into account that single atoms are susceptible to aggregation upon an increase in metal loading, it seems a formidable challenge to prepare a stable, high-atom-density SAC (*e.g.*, 1–5 wt% loading) where all of the metal atoms are isolated.

One feature of SACs which is distinctive from their nanoparticle (NP) counterparts is that the active single atoms are usually positively charged by bonding with more electronegative elements (*e.g.*, oxygen atoms) on the support surface. Considering that alkalis are well known electronic modifiers in transition metal catalysts,^6^ and that they are even able to promote Pt dispersion as single atoms,^7^ we envision that by modifying the high-loading Pt/FeO_*x*_ catalysts with alkalis, one might be able to prepare a high-atom-density SAC, and eventually transform the less chemoselective high-loading Pt/FeO_*x*_ into a highly chemoselective one.

## Results and discussion

First we prepared a series of Pt/FeO_*x*_ catalysts with different Pt loadings using co-precipitation of ferric nitrate (Fe(NO_3_)_3_) and chloroplatinic acid (H_2_PtCl_6_) with ammonia carbonate ((NH_4_)_2_CO_3_) as the precipitating agent (for details see ESI†) and then tested them for hydrogenation of 3-nitrostyrene, one of the most demanding reactions in the chemoselective hydrogenation of nitroarenes. Unless otherwise noted, all of the catalysts were reduced in 10% H_2_/He at 250 °C for 0.5 h prior to the reaction and characterizations. The reaction tests showed that selectivity to 3-aminostyrene decreased with an increase in Pt loading: 0.12% Pt/FeO_*x*_ yielded a selectivity of 97.6% whereas 2.16% Pt/FeO_*x*_ yielded a selectivity of only 66.4% (Table S1†). Apparently, the high selectivity was only achievable with low levels of Pt loading, consistent with previous reports.^2b,5a^ Meanwhile, we found that the precipitating agent greatly affected the selectivity of the resultant catalysts, *e.g.* a relatively high selectivity could be obtained even with a high Pt loading catalyst when Na_2_CO_3_ was used as the precipitating agent (Table S2†). We speculated that the residual Na ions in the catalysts must play a key role in directing the chemoselectivity.

In order to study the effect of Na ions in a controllable manner, we subsequently used the above Na-free 2.16 wt% Pt/FeO_*x*_ as the starting catalyst and introduced varying amounts of sodium with sodium nitrate as the precursor. The resulting catalysts are designated as *y*% Na–2.16% Pt/FeO_*x*_, where *y* refers to the weight content of sodium. The catalytic performances are shown in Table 1. To our delight, the selectivity towards 3-aminostyrene increased monotonically with the Na content until a plateau (∼97%) was reached at 3.3% Na while the activity did not change much with Na content. Further increasing the Na content to 7.6% led to a dramatic decline in activity. The best result was obtained at 5.03% Na, which corresponds to a Na/Pt atomic ratio of 19.8. This catalyst afforded a TOF of 1083 h^–1^ and a selectivity to 3-aminostyrene of 97.5%. This high TOF level is slightly lower than that of the 0.08% Pt/FeO_*x*_ SAC we previously reported,^5*a*^ but the efficiency based on the total weight of catalyst is increased by more than 20 times due to a relatively high Pt loading. This result is very important for practical applications since it suggests that the productivity can be increased by 20 times. To our delight, in comparison with the Pt/TiO_2_,^2*b*^ Pt/ZnO,^2*i*^ Au/TiO_2_,^3*a*^ Ag@CeO_2_^ 3*d*^ and RhIn/SiO_2_^ 2*j*^ catalysts which were earlier reported to be highly chemoselective for the hydrogenation of 3-aminostyrene, our present 5.03% Na–2.16% Pt/FeO_*x*_ catalyst is several-fold to two orders of magnitude more active, based either on per metal or per weight of catalyst (Table S3†). Moreover, the catalyst could be reused several times without a decrease in activity or selectivity (Table S4†), and could tolerate a wide spectrum of substrates with different functional groups, such as triple bonds, ketones, aldehydes, and halogens (Table 2). Like sodium ions, other alkalis such as Li and K ions also showed remarkably positive effects on the chemoselectivity (Table S5†). These results clearly demonstrate that alkali ions are able to transform the less chemoselective high-loading Pt/FeO_*x*_ into a highly chemoselective one.

**Table 1:** The effect of Na content on the chemoselective hydrogenation of 3-nitrostyrene

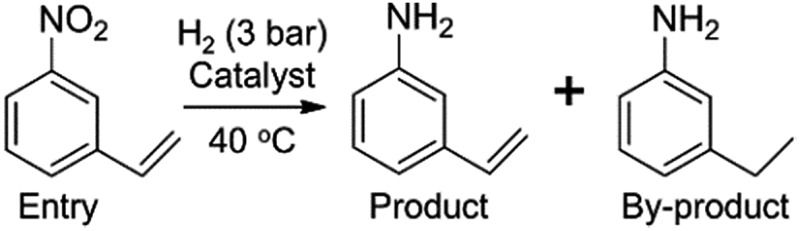				
Catalyst	Na/Pt ratio^*a*^	Time (min)	Conv. (%)	Sel. (%)
2.16% Pt/FeO_*x*_	0	36	98.7	66.4
0.74% Na–2.16% Pt/FeO_*x*_	2.9	35	95.3	67.5
1.75% Na–2.16% Pt/FeO_*x*_	6.9	28	96.9	85.6
2.64% Na–2.16% Pt/FeO_*x*_	10.4	29	94.9	90.5
3.30% Na–2.16% Pt/FeO_*x*_	13.0	26	95.5	96.7
5.03% Na–2.16% Pt/FeO_*x*_	19.8	32	95.1	97.5
7.60% Na–2.16% Pt/FeO_*x*_	30	300	94.2	97.4

^*a*^Atomic ratio. Pretreatment conditions: 5 ml toluene, 1 MPa H_2_, 40 °C, 1 h. Reaction conditions: Pt/substrate = 0.22 mol%, 5 ml reaction mixture, 0.5 mmol substrate, toluene as solvent, *o*-xylene as internal standard.

**Table 2:** Chemoselective hydrogenation of different substituted nitroarenes to the corresponding anilines over the 5.03% Na–2.16% Pt/FeO_*x*_ catalyst^*a*^

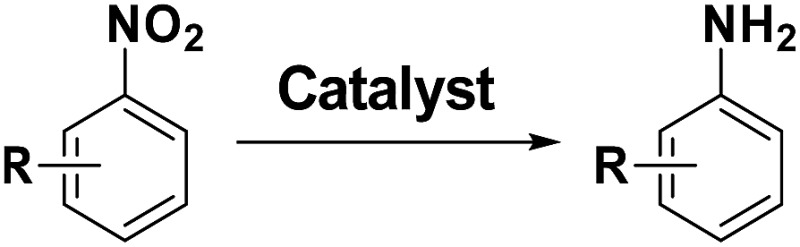				
Entry	Substrate	Product	Time (min)	Yield (%)
1	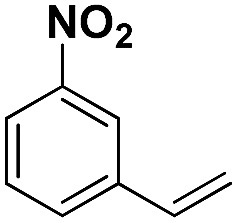	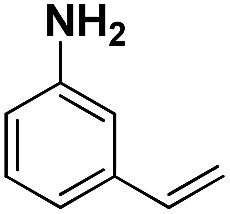	32	92.7
2	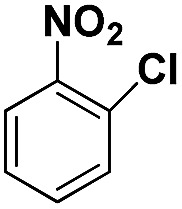	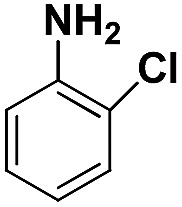	40	97.2
3	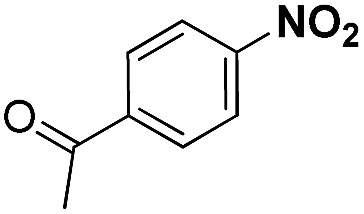	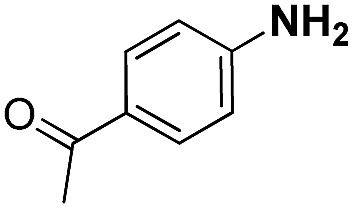	20	96.3
4	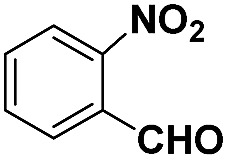	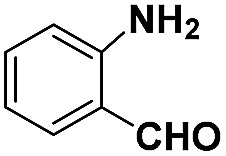	60	94.8
5^*^	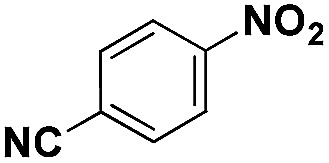	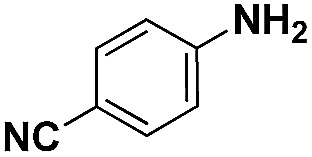	85	95.7
6^+^	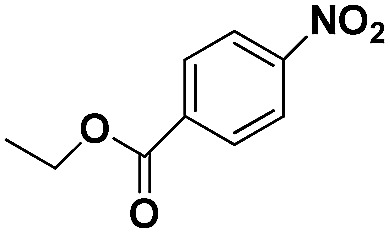	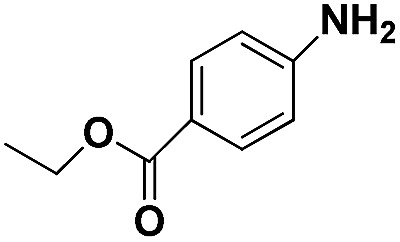	70	96.8

^*a*^Pretreatment conditions: 5 ml toluene, 1 MPa H_2_, 40 °C, 1 h. Reaction conditions: *T* = 40 °C, *P* = 3 bar, Pt/substrate = 0.22 mol%, 5 ml reaction mixture, 0.5 mmol substrate, toluene as solvent. ^*^Pt/substrate = 0.44 mol%, *T* = 50 °C, *P* = 6 bar. ^+^ *T* = 50 °C, *P* = 6 bar.

**Fig. 1:**
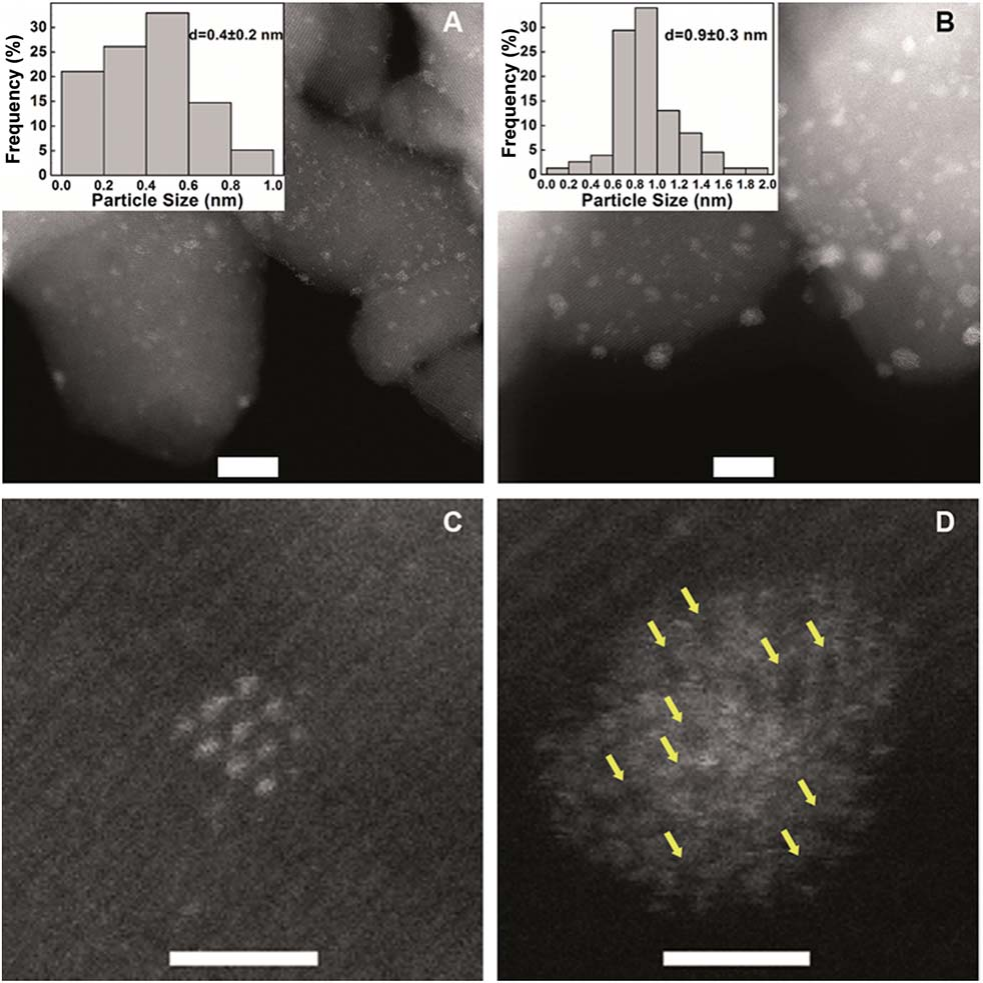
HAADF-STEM images and size distributions of different catalysts. (A) 2.16% Pt/FeO_*x*_; (B) 5.03% Na–2.16% Pt/FeO_*x*_; (C) a Pt particle of 2.16% Pt/FeO_*x*_ and (D) a Pt particle of 5.03% Na–2.16% Pt/FeO_*x*_. In (A) and (B), scale bar = 5nm; in (C) and (D), scale bar = 1nm.

To understand the remarkable effect of alkali ions on the Pt/FeO_*x*_, we performed multifaceted characterizations including X-ray diffraction (XRD), aberration-corrected high-angle annular dark-field scanning transmission electron microscopy (HAADF-STEM), X-ray absorption spectroscopy (XAS), Mössbauer spectroscopy and diffuse reflectance infrared Fourier transform spectroscopy (DRIFT). The XRD patterns of the samples, with or without Na ions, present only Fe_3_O_4_ phase (Fig. S1†). No diffraction peaks of platinum were detected, suggesting that the Pt species is highly dispersed even at a high Pt loading. Fig. 1 and S2† show the HAADF-STEM images of two typical samples, 5.03% Na–2.16% Pt/FeO_*x*_ and 2.16% Pt/FeO_*x*_. At first sight, the Na-containing sample contains much bigger particles than the Na-free sample, and the average particle size in the former is 0.9 nm while it is 0.4 nm in the latter. However, close examination of each particle in the Na-containing sample led to a completely opposite conclusion because the presence of the Na ion greatly changed the morphology of the Pt particles. As shown in Fig. 1D, one can observe apparent dark regions within the particles in the Na-containing sample, which is in contrast with the well-crystallized Pt particles in the Na-free sample (Fig. 1C). Since both Fe and Na are lighter elements (compared to Pt), the presence of either Fe or Na can give such contrasts as seen in the HAADF-STEM images. Examination of many different particles revealed that almost every particle in the Na-containing sample is assembled by loosely and randomly stacked single atoms or clusters of only a few atoms. This can be visualized more clearly in the catalysts reduced at a higher temperature (Fig. S3†). These observations suggest that the particles in the HAADF images may contain Pt atoms as well as Na and/or Fe atoms. The addition of Na ions prevented the formation of Pt crystals even at a relatively high loading of Pt. The XAS data provided further support for this proposed model.

**Fig. 2:**
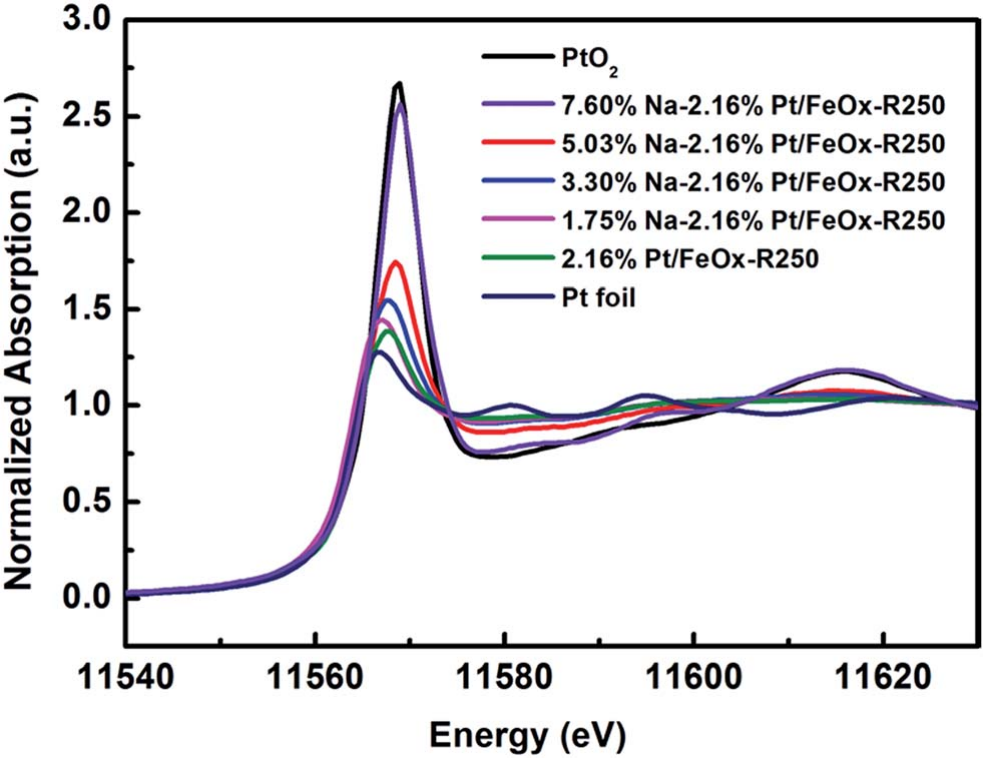
Normalized XANES spectra at the Pt L_III_ edge of 2.16% Pt/FeO_*x*_ catalysts with different Na loadings after reduction.

To uncover the nature of the interactions between the Pt, Na and the Fe_3_O_4_ support, we performed *in situ* XAS experiments during catalyst reduction. In the XANES spectra, the whiteline intensity at the Pt L_III_ edge decreases continuously with an increase in the reduction temperature, with or without Na (Fig. S4†). However, comparing the Na-free and Na-containing samples, one can clearly see that the latter has a higher whiteline intensity than the former at the same reduction temperature (Fig. S5†). Moreover, with an increase in Na content, the whiteline intensity increased monotonically (Fig. 2), indicating that Pt became more positively charged with the addition of Na. Consistently, the temperature programmed reduction of H_2_ (H_2_-TPR) showed that with an increase in the Na content, the PtO_*x*_ species became more difficult to reduce (Fig. S6†). The best-fitted EXAFS results are summarized in Table 3. Compared with the Na-free sample, the Na-containing sample has a larger Pt–O coordination number (2.5 *vs.* 1.2), smaller Pt–Fe and Pt–Pt coordinations (totally 0.9 *vs.* 1.8), and a longer Pt–Pt distance (2.75 *vs.* 2.68 Å), suggesting that the Pt centers in the Na-containing sample are more oxidized, the Pt–Pt coordination is more unsaturated and the Pt–Pt metallic bonding becomes weaker. This result is in good agreement with the conclusion from the HAADF-STEM imaging that the addition of Na greatly limits the formation of Pt crystals. In addition, there is a new Pt–Na contribution at a distance of 2.95 Å with a coordination number of 1.0. Such a long bonding length between Pt and Na can be ascribed to bridge linkage like Pt–O–Na. The formation of a Pt–O–Na interaction was also proved by a control experiment, in which an excess amount of Na was washed away and the residual Na to Pt atomic ratio was close to 1 (0.83 and 0.88, see Table S6†). The reaction test showed that the performance of the catalyst remained almost unchanged after such washing treatment (Table S7†), indicating that the effective Na/Pt atomic ratio was roughly 1/1.

**Table 3:** EXAFS data fitting results of *y*% Na–2.16% Pt/FeO_*x*_ samples^*a*^

Samples	Shell	*N*	*R* (Å)	*σ* ^2^ × 10^2^ (Å^2^)	Δ*E*_0_ (eV)	*r*-factor (%)
Pt foil	Pt–Pt	12.0	2.76	0.41	6.1	0.42
PtO_2_	Pt–O	6.0	2.00	0.41	7.2	0.56
	Pt–Pt	6.0	3.08	0.99	2.1	
2.16% Pt/FeO_*x*_	Pt–O	1.2	1.99	0.30	4.7	0.91
	Pt–Fe	1.1	2.54	0.54	4.7	
	Pt–Pt	0.7	2.68	0.54	4.7	
5.03% Na-2.16% Pt/FeO_*x*_	Pt–O	2.5	2.01	0.18	9.6	0.92
	Pt–Fe	0.4	2.54	0.46	9.6	
	Pt–Pt	0.5	2.75	0.46	9.6	
	Pt–Na	1.0	2.95	0.73	9.6	

^*a*^ *N*, the coordination number for the absorber–backscatterer pair. *R*, the average absorber–backscatterer distance. *σ* ^2^, the Debye–Waller factor. Δ*E*_0_, the inner potential correction. The accuracies of the above parameters were estimated as *N*, ±20%; *R*, ±1%; *σ* ^2^, ±20%; Δ*E*_0,_ ±20%. The data ranges used for data fitting in *k*-space (Δ*k*) and *R*-space (Δ*R*) are 3.0–10.7 Å^–1^ and 1.2–3.5 Å, respectively.

**Fig. 3:**
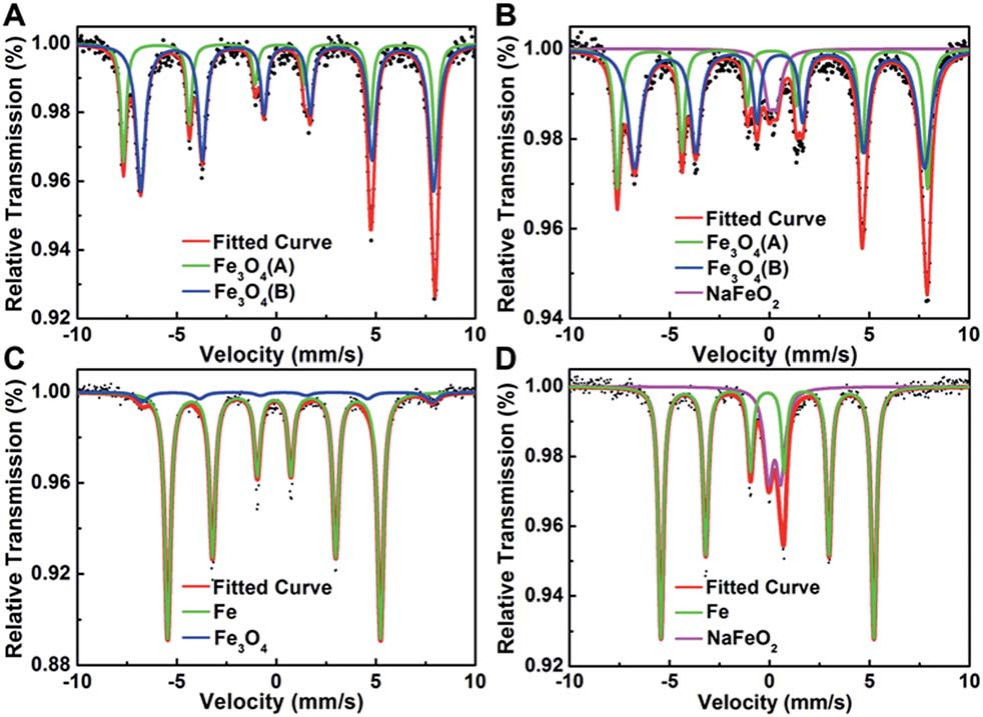
Mössbauer spectroscopy of (A) 2.16% Pt/FeO_*x*_-R250; (B) 5.03% Na-2.16% Pt/FeO_*x*_-R250; (C) 2.16% Pt/FeO_*x*_-R400 and (D) 5.03% Na–2.16% Pt/FeO_*x*_-R400 catalysts.

Considering that Pt interacts with Na ions *via* oxygen atoms on the Fe_3_O_4_ support, we then performed ^57^Fe Mössbauer spectroscopy to probe the local environment of iron oxides surrounding the Pt species. As shown in Fig. 3 and Table S8,† compared with the Na-free sample which is characterized with two sextets of magnetite, there is a new and well-defined doublet with an isomer shift value of 0.26 mm s^–1^ and a quadrupole splitting value of 0.42 mm s^–1^ in the Na-containing sample, which can be assigned to the high-spin Fe^3+^ of NaFeO_2_.^8^ Moreover, the amount of NaFeO_2_ species became more pronounced when the reduction temperature was raised to 400 °C, at which all the magnetite was reduced to metallic Fe while Pt was still positively charged (Fig. 3 and S5†). These results lead us to deduce that the cationic Pt must arise from the linkage to NaFeO_2_, otherwise Pt would be reduced to its metallic state at such elevated temperatures. Therefore, we propose that Pt interacts with the NaFeO_2_ surface species *via* bridging oxygen, forming a Pt–O–Na–O–Fe-species, which creates low-coordinated, or even isolated, positively charged Pt centers to ensure both high activity and chemoselectivity for nitroarene hydrogenation. The particles with unusual contrast observed in the HAADF-STEM images of the Na-containing sample are most probably assemblies of the Pt–O–Na–O–Fe-species.

To further prove the above-proposed model that the surface NaFeO_2_ species interacts with Pt and thus enhances its dispersion, we prepared a sandwich catalyst composed of Pt/NaFeO_2_/FeO_*x*_, by first forming a layer of NaFeO_2_ on the FeO_*x*_ support and then depositing the Pt species (for details see ESI†). The NaFeO_2_/FeO_*x*_ structure was identified by Mössbauer spectroscopy (Fig. S7 and Table S9†). The HAADF-STEM images of this sample clearly show the lattice fringes of the Fe_3_O_4_ nanocrystal, the amorphous layer of NaFeO_2_ on the surface of Fe_3_O_4_, and the assemblies of Pt single atoms preferentially deposited on the NaFeO_2_ surface layer (Fig. S8†). The assemblies of Pt single atoms do not form Pt crystals due to the interaction with NaFeO_2_, which is consistent with the series of *y*% Na–2.16% Pt/FeO_*x*_ catalysts. Interestingly, this Pt/NaFeO_2_/FeO_*x*_ catalyst exhibited 99.3% selectivity towards 3-aminostyrene at full conversion of the substrate (Table S10†). Such an exceptionally high selectivity at full conversion of substrate has never been obtained on any Pt catalyst.

**Fig. 4:**
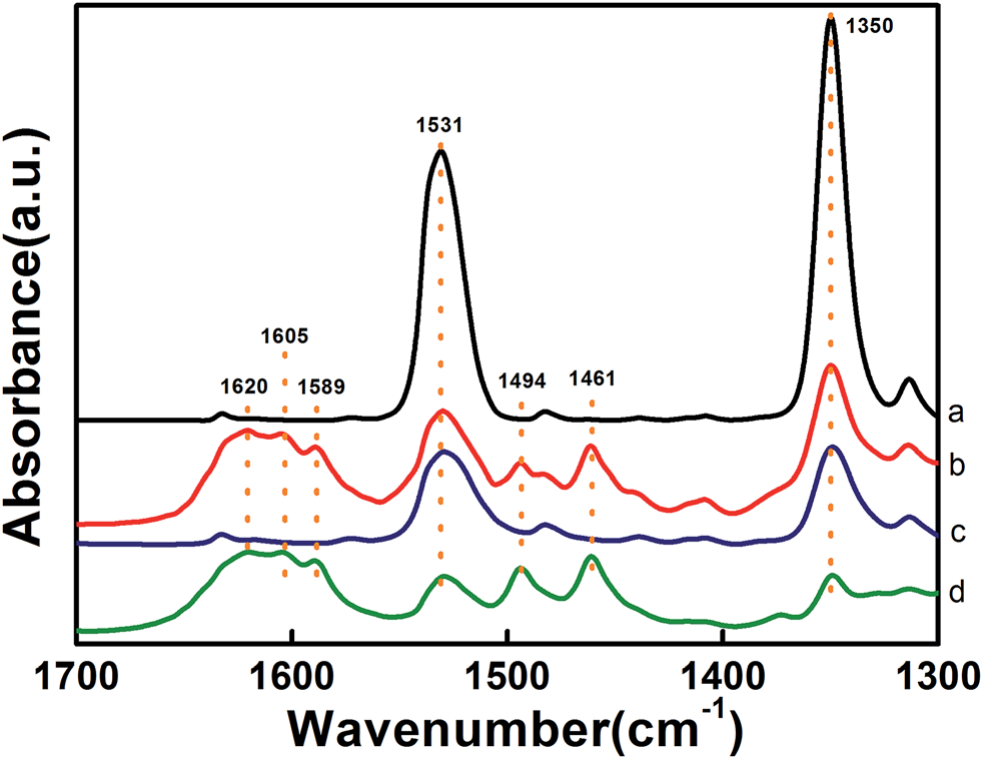
DRIFT spectra of 3-nitrostyrene adsorption at 80 °C on (a and b) 5.03% Na–2.16% Pt/FeO_*x*_-R250 and (c and d) 2.16% Pt/FeO_*x*_-R250 before (a and c) and after (b and d) introduction of H_2_.

The formation of the NaFeO_2_ surface layer not only promotes the dispersion of Pt but also has a significant impact on the preferential adsorption of the nitro group. As shown in the DRIFT spectra in Fig. 4, the adsorption of 3-nitrostyrene produced two bands at 1531 cm^–1^ and 1350 cm^–1^, which are attributed to asymmetric stretching (ν_as_) and symmetric stretching (ν_ss_) vibrations of the nitro group,^9a,9b^ respectively. Comparing the DRIFT spectra of different catalysts, one can clearly see that the 5.03% Na–2.16% Pt/FeO_*x*_ catalyst adsorbs the nitro group much more strongly than the 2.16% Pt/FeO_*x*_ catalyst does, suggesting that the presence of the Na cation intensifies the adsorption of the nitro group. A control experiment with the Na-modified FeO_*x*_ support reveals that the nitro group adsorbs on the support, rather than on the Pt species (Fig. S9†). Introduction of H_2_ to the 2.16% Pt/FeO_*x*_ and 5.03% Na–2.16% Pt/FeO_*x*_ samples which were allowed to pre-adsorb 3-nitrostyrene led to the appearance of new bands at 1589 cm^–1^ (–NO),^9c,9d^ 1461 cm^–1^ (–NOH),^9*b*^ 1620 cm^–1^, 1605 cm^–1^ and 1494 cm^–1^ (–NH_2_).^9c,9e^ These were concurrent with sharp decreases in the intensities of the nitro absorption bands, demonstrating the stepwise reduction from a nitro group to an amino group. In contrast, the same procedure on the FeO_*x*_ support and –modified FeO_*x*_ support did not bring about any change in the nitro adsorption bands (Fig. S9†). Taking these results together, we can conclude that the nitro group is adsorbed on the NaFeO_2_ surface while H_2_ is activated on the Pt species, and that the hydrogenation reaction occurs most likely on the interface between the Pt and the NaFeO_2_. The formation of the NaFeO_2_ species makes the nitro group strongly adsorb on it, thereby increasing the chemoselectivity of the nitro group hydrogenation.

Control experiments using nitrobenzene and styrene as the reaction substrates verify that the competitive adsorption of –NO_2_ over the C=C group on the catalyst governs the chemoselectivity. As shown in Table S11,† under non-competitive reaction conditions (only one functional group was present), the presence of Na led to the reaction rate for C=C hydrogenation being reduced by more than 65%, while the rate of nitrobenzene hydrogenation was reduced by only 26%. This demonstrates that the Pt–O–Na–O–Fe-species is intrinsically selective for –NO_2_ hydrogenation, which is quite different from the Na-free sample that is almost non-selective. A more striking difference was observed under competitive reaction conditions (both functional groups were present): the rates for nitrobenzene hydrogenation were almost the same for both catalysts while that of styrene hydrogenation over the Na-containing catalyst was reduced by one order of magnitude compared to the Na-free catalyst, thus greatly enhancing the chemoselectivity for –NO_2_ hydrogenation.

**Fig. 5:**
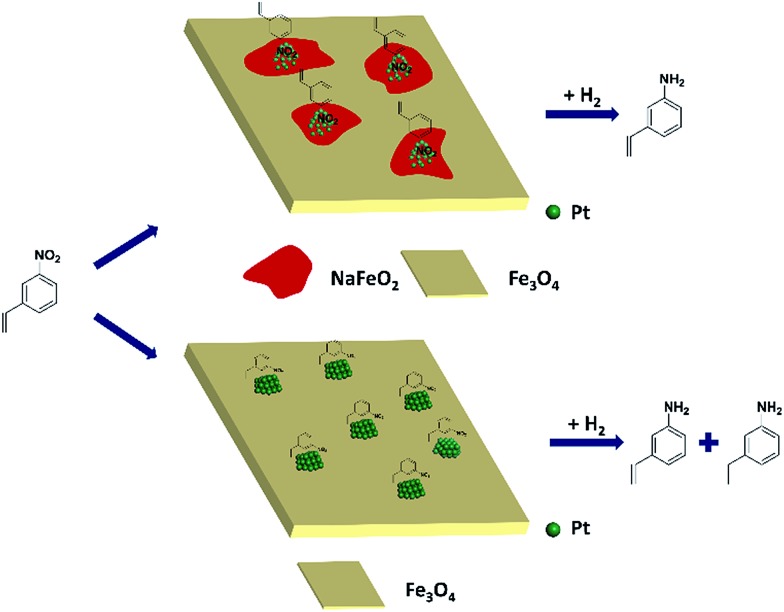
The proposed model for chemoselective hydrogenation of 3-nitrostyrene over Na-modified Pt/FeO_*x*_ catalysts.

Based on the above results, it can be reasonably concluded that it was the surface layer of NaFeO_2_ that directed the formation of pseudo-single atoms of Pt, by which the C=C adsorption was almost completely suppressed while the –NO_2_ group was adsorbed preferentially, eventually leading to the chemoselective hydrogenation of the nitro group, as illustrated in Fig. 5.

## Conclusions

In conclusion, we have found that alkali metal cations are able to transform a less chemoselective high-loading Pt/FeO_*x*_ catalyst to a highly chemoselective one without compromising the activity. Na cations interact with the Pt/FeO_*x*_ catalyst to form the Pt–O–Na–O–Fe surface species, which not only prevents the formation of Pt crystals, but also provides low-coordinated or even isolated and positively charged Pt centers. Such a Pt–O–Na–O–Fe species is intrinsically more selective for –NO_2_ hydrogenation than for C=C hydrogenation. To the best of our knowledge, this is the first report in which both high activity and chemoselectivity are achieved with a high loading transition metal catalyst. This strategy for engineering the electronic and geometric properties of Pt by doping alkali metals can be extended to other supported metal catalysts for various chemical reactions.
