# Supplementary material for: Remarkable effect of alkalis on the chemoselective hydrogenation of functionalized nitroarenes over high-loading Pt/FeO x catalysts
Source: Chem Sci. 2017 May 16;8(7):5126–31. doi: 10.1039/c7sc00568g (PMC5615215; doi:10.1039/c7sc00568g)
Supplement: Supplementary file 1 [file SC-008-C7SC00568G-s001.pdf]

Electronic Supplementary Material (ESI) for Chemical Science.
This journal is © The Royal Society of Chemistry 2017

# Supporting Information

# Remarkable Effect of Alkalis on Chemoselective Hydrogenation of Functionalized Nitroarenes over High-Loading Pt/FeOx Catalysts

**Haisheng Wei,[a] Yujing Ren,[a,b] Aiqin Wang,*[a] Xiaoyan Liu,[a] Xin Liu,[a] Leilei Zhang,[a] Shu Miao,[a] Lin Li,[a] Jingyue Liu,[c] Junhu Wang,[a] Guofu Wang,[d] Dangsheng Su,[a] Tao Zhang*[a]**

**[a] State Key Laboratory of Catalysis, *i*ChEM, Dalian Institute of Chemical Physics, Chinese Academy of Sciences, Dalian, 116023, China.**

**[b] University of Chinese Academy of Sciences, Beijing, 100049, China.**

**[c] Department of Physics, Arizona State University, Tempe, Arizona, 85287, USA.**

**[d] State Key Laboratory of Coal Conversion, Institute of Coal Chemistry, Chinese Academy of Sciences, Taiyuan, 030001, China.**

***To whom correspondence should be addressed.**

**aqwang@dicp.ac.cn**

**taozhang@dicp.ac.cn**

## Experimental details

## Preparation of catalysts

The Pt/FeOx catalysts with different Pt loadings were prepared by co-precipitation of an aqueous solution of chloroplatinic acid precursor ($H_2PtCl_6 \cdot 6H_2O$, 7.59×$10^{-2}$ mol $l^{-1}$) and 20 ml of an aqueous solution of ferric nitrate precursor ($Fe(NO_3)_3 \cdot 9H_2O$, 1 mol $l^{-1}$), with ammonia carbonate as the precipitating agent. The process was conducted at 50 $^{o}$C and the pH value of ~ 8. After stirring for 3 h and aging for 3 h, the solid was recovered by filtration and washing, dried at 60 $^{o}$C overnight, and calcined at 400 $^{o}$C for 5 h. The y%Na-2.16%Pt/FeOx catalysts with different Na loadings were prepared by incipient wetness impregnation of the dried 2.16%Pt/FeOx sample with an appropriate amount of $NaNO_3$ solution and calcined at 400 $^{o}$C for 5 h. Before characterizations and activity tests, all the samples were reduced in 10% $H_2$/He at 250 $^{o}$C or 400 $^{o}$C for 0.5 h.

The $NaFeO_2$/FeOx support was prepared by precipitation of an aqueous solution of ferric nitrate precursor ($Fe(NO_3)_3 \cdot 9H_2O$, 1 mol $l^{-1}$) with ammonia carbonate. The process was conducted at 50 $^{o}$C and the pH value of ~ 8. After stirring for 3 h and aging for 3 h, the solid was recovered by filtration and washing, dried at 60 $^{o}$C overnight. Then the acquired solid was impregnated by an appropriate amount of $NaNO_3$ solution, calcined at 400 $^{o}$C for 5 h and reduced at 400 $^{o}$C for 0.5 h. Next the 0.74%Pt/ $NaFeO_2$/FeOx was prepared by a deposition-precipitation of a mixture of an aqueous solution of chloroplatinic acid precursor ($H_2PtCl_6 \cdot 6H_2O$, 7.59×$10^{-2}$ mol $l^{-1}$) and previous $NaFeO_2$/FeOx support with ammonia carbonate as the precipitating agent. The process was conducted at 80 $^{o}$C and the pH value of ~ 8. After stirring for 6 h, the solid was recovered by filtration and washing, dried at 60 $^{o}$C overnight. Before characterizations and activity tests, the 0.74%Pt/ $NaFeO_2$/FeOx was reduced in 10% $H_2$/He at 250 $^{o}$C for 0.5 h.

## Characterization methods

Metal loadings in the catalyst samples were determined by inductively coupled plasma spectroscopy (ICP-AES) on an IRIS Intrepid II XSP instrument (Thermo Electron Corporation).

X-ray diffraction (XRD) analysis was performed on a PANalytical X' pert diffractometer with a Cu-Ka radiation source (40 kV and 40 mA). A continuous mode was used to record data in the 2θ range from 10° to 80°.

Temperature-programmed reduction of hydrogen ($H_2$-TPR) was performed on an Auto Chem II 2920 apparatus. For each measurement, 100 mg of a catalyst sample was loaded into a U-shape quartz reactor and pretreated at 150 $^{o}$C in Ar for 0.5 h to remove adsorbed carbonates and hydrates. After the sample was cooled down to room temperature, the flowing gas was switched to 10 vol% $H_2$/Ar and the sample was heated to 800 $^{o}$C with a temperature ramp of 10 $^{o}$C $min^{-1}$.

Aberration-corrected high-angle annual dark-filed scanning transmission electron microscopy (HAADF-STEM) images were obtained on a JEOL JEM-ARM200F equipped with a CEOS probe corrector, with a guaranteed resolution of 0.08 nm. Before microscopy examination, the sample was ultrasonically dispersed in ethanol for 5-10 minutes, and then a drop of the suspension was dropped on a copper TEM grid coated with a thin holey carbon film.

The X-ray absorption spectra (XAS) including X-ray absorption near edge structure (XANES) and extended X-ray absorption fine structure (EXAFS) at Pt $L_{III}$-edge of the samples were measured at the beamline 14W of Shanghai Synchrotron Radiation Facility (SSRF) in China. The output beam was selected by Si(111) monochromator. The energy was calibrated by the Pt foil.

Before measurement, the samples were reduced under 10%$H_2$/He at 250 °C for 0.5 h, followed by purging with pure He until cooled to room temperature. Next, the samples were evacuated and transferred to glove box without exposure to air. The sample was sealed with Kapton films in the glove box before XAFS measurement. The data were collected at room temperature under fluorescence mode by using solid state detector. For in-situ XANES measurements, the samples were diluted with boron nitride and then tableted. The tablets were placed in the in-situ cell, sealed by Kapton film and O-ring and reduced under 20%$H_2$/He with a programmed heating procedure: with a temperature ramp of 10 °C $min^{-1}$ from 20 °C to 200 °C, and 3.125 °C $min^{-1}$ from 200 °C to 400 °C. Meanwhile, the data were collected under fluorescence mode by using a solid state detector. Athena software package was employed to process the XAS data.

Mössbauer spectra were recorded in the transmission geometry at room temperature using a 25mCi $^{57}$Co/Rh-matrix source in constant acceleration mode. The isomer shift is referred to α-Fe at room temperature. Before measurement, the samples were reduced under 10%$H_2$/He at 250 °C for 0.5 h, followed by purging with pure He until cooled to room temperature. Next, the samples were evacuated and transferred into the sample cell sealed by tape in glove box without exposure to air. The data were collected at room temperature under transmission mode. Spectra were fitted to Lorentzian curves with the mosswinn software.

In-situ diffuse reflectance infrared Fourier transform spectra (DRIFT) were collected at 80 °C with a Bruker Vertex 70 spectrometer equipped with a mercury cadmium telluride (MCT) detector using 8 and 16 scans at a resolution of 4 $cm^{-1}$. In order to compare the difference of 3-nitrostyrene absorption between Na-free and Na-containing samples, 35mg of each sample was put in a furnace and reduced in situ at 250 °C and 400 °C with 20 ml/min 10% $H_2$/He for 0.5 h, respectively, cooled to 80 °C in helium, and background spectrum was recorded. Then, with the protection of pure helium, a fixed amount (5ul) of 3-nitrostyrene was introduced to the sample for adsorption, and the spectra were collected till the steady state. Then pure hydrogen was introduced to the sample, and the spectra of 3-nitrostyrene hydrogenation process were collected.

## Reaction tests

The chemoselective hydrogenation of 3-nitrostyrene was performed in an autoclave equipped with pressure control system. Before the reaction test, the catalyst was pretreated by the following steps: First the powder catalyst, 5 ml toluene and magnetic bar were put into the autoclave. And then the autoclave was charged with 1 MPa hydrogen and sealed. Finally the reactor was heated at 40 °C for 1 h in the wash bath to allow for the reduction treatment of the catalyst. Subsequently, toluene was poured out and a total volume of 5 ml solution including 3-nitrostyrene, toluene and internal standard O-xylene, was put into the autoclave. Afterwards, the autoclave was purged by flushing with 10 bar hydrogen for five times. After that, the autoclave was pressurized with hydrogen to 3 bar, then it was heated to 40 °C in a water bath with a magnetic stirrer to initiate the reaction. During the reaction the $H_2$ pressure decreased gradually as a result of hydrogenation. After reaction, the product was analyzed by gas chromatography–mass spectrometry (GC-MS).

**Table S1.** Chemoselective hydrogenation of 3-nitrostyrene over Pt/FeOx catalysts prepared by $(NH_4)_2CO_3$ with different Pt loadings.

| Catalyst | Time(min) | Conv. (%) | Sel. (%) |
|---|---|---|---|
| 0.12%Pt/FeOx[a] | 44 | 96.3 | 97.6 |
| 0.48%Pt/FeOx | 23 | 99.3 | 95.1 |
| 0.93%Pt/FeOx | 27 | 95.4 | 92.5 |
| 1.67%Pt/FeOx | 15 | 93.7 | 85.6 |
| 2.16%Pt/FeOx | 36 | 98.7 | 66.4 |

Pretreatment condition: 5 ml toluene, 1 MPa $H_2$, 40 $^oC$, 1 h.

Reaction conditions: T=40 $^oC$, P=3 bar, Pt/substrate = 0.22 mol%; [a] Pt/substrate = 0.12 mol%.5 ml reaction mixture: 0.5 mmol substrate, toluene as solvent, o-xylene as internal standard.

**Table S2.** Chemoselective hydrogenation of 3-nitrostyrene over Pt/FeOx catalysts prepared by $Na_2CO_3$ with different Pt loadings

| Catalyst | Na (wt%) | Na/Pt ratio[a] | Time(min) | Conv. (%) | Sel. (%) |
|---|---|---|---|---|---|
| 0.08%Pt/FeOx | 0.40 | 42.4 | 50 | 96.5 | 98.6 |
| 0.31%Pt/FeOx | 0.49 | 13.4 | 67 | 95.8 | 94.4 |
| 0.75%Pt/FeOx | 0.13 | 1.5 | 73 | 96.7 | 92.6 |
| 4.3%Pt/FeOx[b] | 0.74 | 1.5 | 34 | 94.2 | 92.7 |

[a] atomic ratio. Pretreatment condition: 5 ml toluene, 1 MPa $H_2$, 40 $^oC$, 1 h.

Reaction conditions: T=40 $^oC$, P=3 bar, Pt/substrate = 0.08 mol%; [a] Pt/substrate = 0.45 mol%.5 ml reaction mixture: 0.5 mmol substrate, toluene as solvent, o-xylene as internal standard.

**Table S3** Comparison of the performances of various catalysts for the hydrogenation of 3-nitrostyrene to 3-vinylaniline.

| Cat. | Temp. ($^oC$) | P (bar) | Time (min) | Conv. (%) | Sel. (%) | TOF ($mol_{conv.}h^{-1}mol_{Pt}^{-1}$) | ($mol_{conv.}h^{-1}g_{cat}^{-1}$) | Ref. |
|---|---|---|---|---|---|---|---|---|
| Na-Pt/FeOx | 40 | 3 | 32 | 95.1 | 97.5 | 1083 | 0.12 | This work |
| Pt/FeOx | 40 | 3 | 50 | 96.5 | 98.6 | 1514 | 0.0062 | 1 |
| $Pt/TiO_2$ | 40 | 3 | 390 | 95.1 | 93.1 | 60 | 0.00062 | 2 |
| Pt/ZnO | 75 | 10 | - | - | 97 | 12.4 | 0.00089 | 3 |
| $Au/TiO_2$ | 120 | 8 | 360 | 98.5 | 95.9 | 173 | 0.013 | 4 |
| $Ag@CeO_2$ | 110 | 6 | 360 | 99 | 99 | 4.85 | 0.018 | 5 |
| $RhIn/SiO_2$ | 75 | 1 | 120 | 99 | 91 | 26.1 | 0.0076 | 6 |

1. H. Wei, X. Liu, A. Wang, L. Zhang, B. Qiao, X. Yang, Y. Huang, S. Miao, J. Liu and T. Zhang, *Nat. Commun.*, 2014, **5**, 5634.

2. A. Corma, P. Serna, P. Concepción and J. C. José, *J. Am. Chem. Soc.*, 2008, **130**, 8748.

3. C. Berguerand, A. Yarulin, F. Cárdenas-Lizana, J. Wärnå, E. Sulman, D. Y. Murzin and L. Kiwi-Minsker, *Ind. Eng. Chem. Res.*, 2015, **54**, 8659.

4. A. Corma and P. Serna, *Science*, 2006, **313**, 332.

5. T. Mitsudome, Y.Mikami, M. Matoba, T. Mizugaki, K. Jitsukawa and K. Kaneda, *Angew. Chem., Int. Ed.*, 2012, **51**, 136.

6. S. Furukawa, K. Takahashi and T. Komatsu, *Chem. Sci.*, 2016, **7**, 4476.

**Table S4.** Recycling results for chemoselective hydrogenation of 3-nitrostyrene over 5.03%Na-2.16%Pt/FeOx catalyst.

| Run times | Time(min) | Conv. (%) | Sel. (%) |
|---|---|---|---|
| $1^{st}$ | 14 | 96.9 | 98.8 |
| $2^{nd}$ | 16 | 95.6 | 98.9 |
| $3^{rd}$ | 17 | 96.8 | 99.0 |
| $4^{th}$ | 23 | 94.9 | 98.7 |

Pretreatment condition: 5 ml toluene, 1 MPa $H_2$, 40 $^oC$, 1 h.

Reaction conditions: T = 40 $^oC$, P = 3 bar, Pt/substrate = 0.44 mol%. 5 ml reaction mixture: 0.5 mmol substrate, toluene as solvent, o-xylene as internal standard.

**Table S5.** Chemoselective hydrogenation of 3-nitrostyrene over 5%Li-1.95%Pt/FeOx and 5%K-1.95%Pt/FeOx catalysts.

| Catalyst | Time(min) | Conv. (%) | Sel. (%) |
|---|---|---|---|
| 5%Li-1.95%Pt/FeOx | 39 | 95.7 | 95.0 |
| 5%K-1.95%Pt/FeOx | 31 | 99.8 | 94.2 |

Pretreatment condition: 5 ml toluene, 1 MPa $H_2$, 40 $^oC$, 1 h.

Reaction conditions: T = 40 $^oC$, P = 3 bar, Pt/substrate = 0.20 mol%. 5 ml reaction mixture: 0.5 mmol substrate, toluene as solvent, o-xylene as internal standard.

**Table S6**. Na content before and after wash experiments.

| Catalyst | Before wash | Wash time (min) | After wash | Na/Pt (atomic ratio) |
|---|---|---|---|---|
| 5.5%Na-1.95%Pt/FeOx-R250 | 5.5% | 30 | 0.19% | 0.83 |
| 5.8%Na-1.93%Pt/FeOx-R250 | 5.8% | 60 | 0.20% | 0.88 |
| 5.8%Na-1.93%Pt/FeOx-R250 | 5.8% | 90 | 0.20% | 0.88 |
| 5.8%Na-1.93%Pt/FeOx-R250 | 5.8% | 120 | 0.20% | 0.88 |

Wash experiment condition: T=90 $^oC$

**Table S7.** Catalytic performance of 5%Na-1.95%Pt/FeOx before and after washing Na.

| Catalyst | Time(min) | Conv. (%) | Sel. (%) |
|---|---|---|---|
| 5.5%Na-1.95%Pt/FeOx | 43 | 99.6 | 95.2 |
| 5.5%Na-1.95%Pt/FeOx-wash | 43 | 98.1 | 93.4 |

Pretreatment condition: 5 ml toluene, 1 MPa $H_2$, 40 $^oC$, 1 h.

Reaction conditions: T=40 $^oC$, P=3 bar, Pt/substrate = 0.20 mol%. 5 ml reaction mixture: 0.5 mmol substrate, toluene as solvent, o-xylene as internal standard.

**Table S8.** Room temperature Mössbauer parameters of 2.16%Pt/FeOx and 5.03%Na-2.16%Pt/FeOx catalysts.

| Catalyst | Mössbauer parameters | | | | |
|---|---|---|---|---|---|
| | IS (mm/s) | QS (mm/s) | H (T) | A (%) | Site |
| 2.16%Pt/FeOx-R250 | 0.28 | -0.01 | 48.6 | 32.3 | $Fe_3O_4$ (A) |
| | 0.65 | 0.01 | 45.7 | 67.7 | $Fe_3O_4$ (B) |
| 5.03%Na-2.16%Pt/FeOx-R250 | 0.25 | 0.02 | 48.3 | 32.4 | $Fe_3O_4$ (A) |
| | 0.61 | 0.00 | 45.0 | 59.0 | $Fe_3O_4$ (B) |
| | 0.26 | 0.42 | - | 8.5 | $NaFeO_2$ |
| 2.16%Pt/FeOx-R400 | 0.00 | 0.00 | 33.2 | 93 | Fe |
| | 0.57 | 0.20 | 45.2 | 7 | $Fe_3O_4$ |
| 5.03%Na-2.16%Pt/FeOx-R400 | 0.00 | 0.00 | 33.0 | 77 | Fe |
| | 0.37 | 0.61 | - | 23 | $NaFeO_2$ |

Mössbauer parameters: IS, isomer shift referred to α-Fe at room temperature; QS, quadrupole splitting; H, hyperfine magnetic field; A, relative area of each site.

**Table S9.** Room temperature Mössbauer parameters of $NaFeO_2$/FeOx support.

| Support | Mössbauer parameters | | | | |
|---|---|---|---|---|---|
| | IS (mm/s) | QS (mm/s) | H (T) | A (%) | Site |
| $NaFeO_2$/FeOx | 0.28 | -0.01 | 48.9 | 26.2 | $Fe_3O_4$(A) |
| | 0.67 | 0.01 | 45.9 | 42.2 | $Fe_3O_4$(B) |
| | 0.99 | 1.06 | - | 18.6 | FeO |
| | 0.30 | 0.67 | - | 13 | $NaFeO_2$ |

Mössbauer parameters: IS, isomer shift referred to α-Fe at room temperature; QS, quadrupole splitting; H, hyperfine magnetic field; A, relative area of each site.

**Table S10.** Chemoselective hydrogenation of 3-nitrostyrene over 0.74%Pt/$NaFeO_2$/FeOx catalyst.

| Catalyst | Time(min) | Conv. (%) | Sel. (%) |
|---|---|---|---|
| 0.74%Pt/$NaFeO_2$/FeOx | 44 | 99.9 | 99.3 |

Pretreatment condition: 5 ml toluene, 1 MPa $H_2$, 40 $^oC$, 1 h.

Reaction conditions: T = 40 $^oC$, P = 3 bar, Pt/substrate = 0.09 mol%. 5 ml reaction mixture: 0.5 mmol substrate, toluene as solvent, o-xylene as internal standard.

**Table S11.** Control experiments with styrene and nitrobenzene as the substrates over 2.16%Pt/FeOx and 5.03%Na-2.16%Pt/FeOx catalysts.

| Catalyst | TOF (mol converted $h^{-1}$ $mol^{-1}$Pt) | | | | Con.[c] | Sel.[c] |
|---|---|---|---|---|---|---|
| | styrene[a] | nitrobenzene[a] | styrene[b] | nitrobenzene[b] | (%) | (%) |
| No Na | 876 | 812 | 147 | 1038 | 98.7 | 66.4 |
| 5.0%Na | 256 | 596 | 20 | 948 | 95.1 | 97.5 |

Pretreatment condition: 5 ml toluene, 1 MPa $H_2$, 40 $^oC$, 1 h.

Reaction conditions: T=40 $^oC$, P=3 bar, Pt/substrate = 0.22 mol%, 5ml toluene, mesitylene was used as internal standard. [a] noncompetitive reaction conditions: 0.5 mmol nitrobenzene or styrene; [b] Competitive reaction conditions: 0.25 mmol nitrobenzene and 0.25 mmol styrene. [c] The values were measured at 36 min for Na-free sample and 32 min for Na-containing sample.

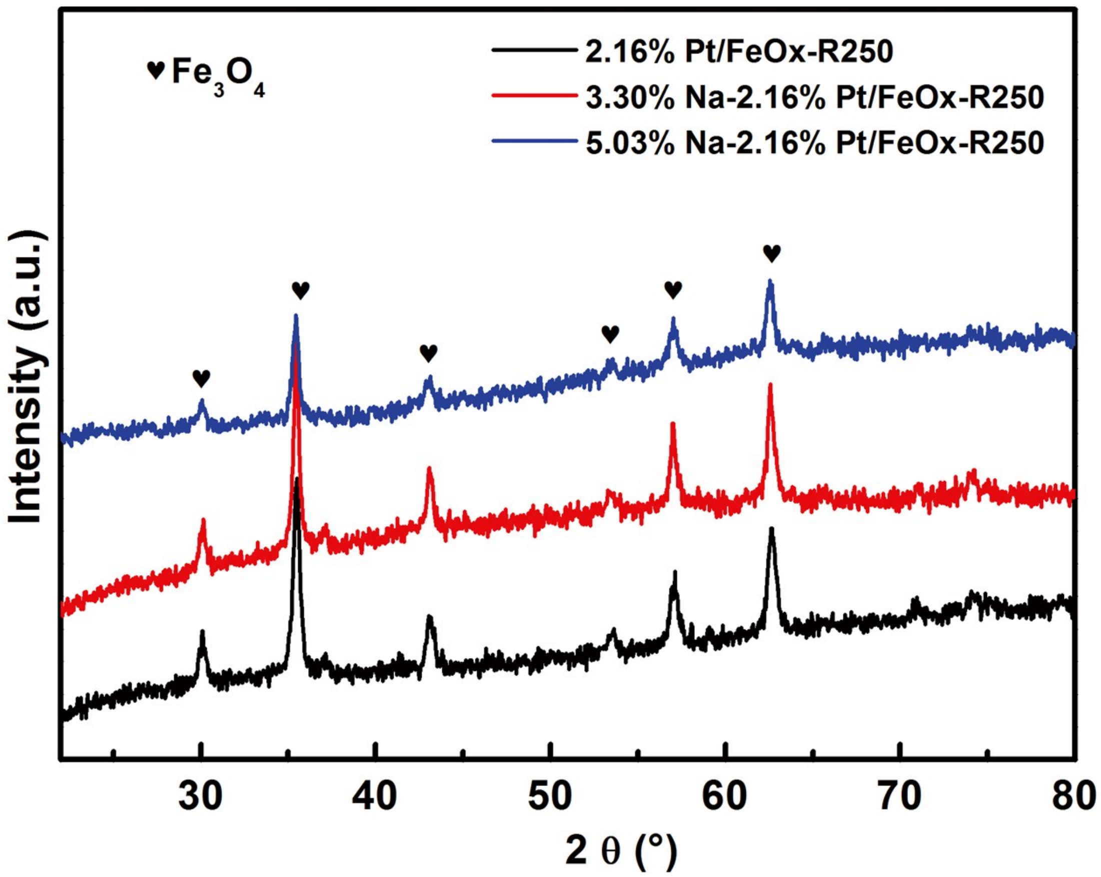


**Figure S1.** XRD patterns of Pt/FeOx catalysts with different Na loadings.

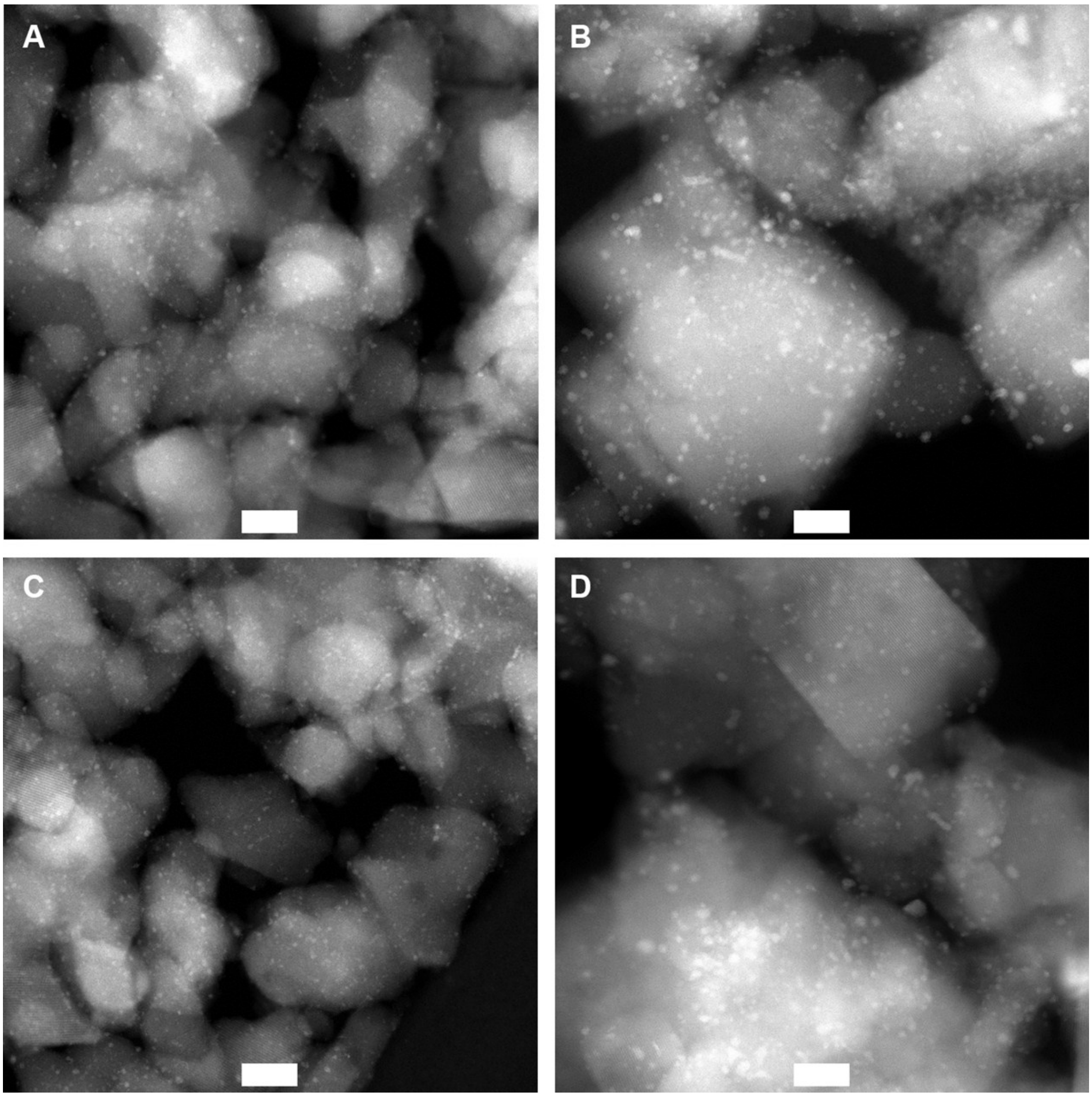


**Figure S2.** HAADF-STEM images of different catalysts. (A, C) 2.16%Pt/FeOx; (B, D) 5.03%Na-2.16%Pt/FeOx. All scale bars are 10 nm.

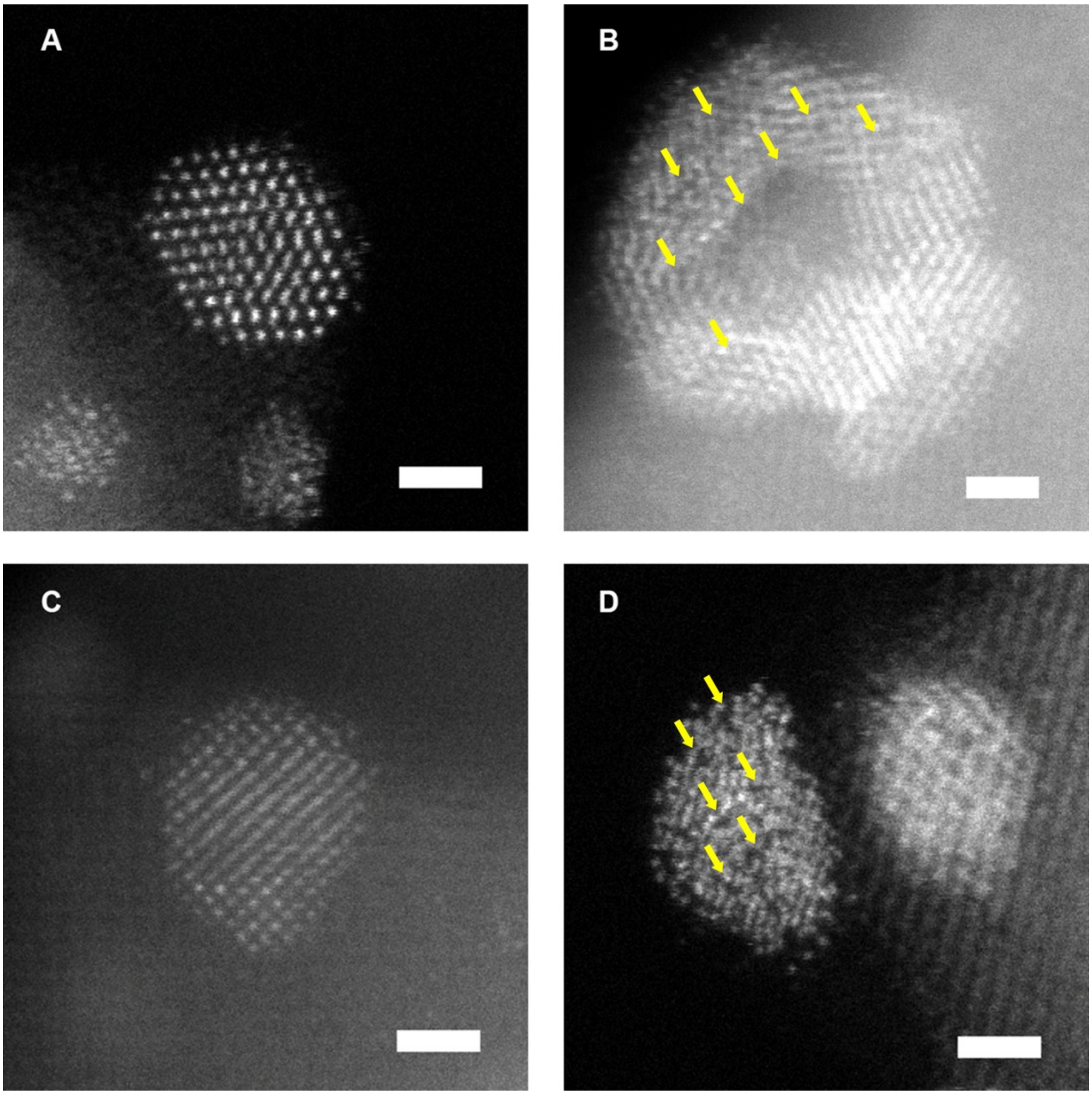


**Figure S3.** HAADF-STEM images of Pt particles in different catalysts.
(A, C) 2.16%Pt/FeOx-R400; (B, D) 5.03%Na-2.16%Pt/FeOx-R400; All scale bars are 1 nm.

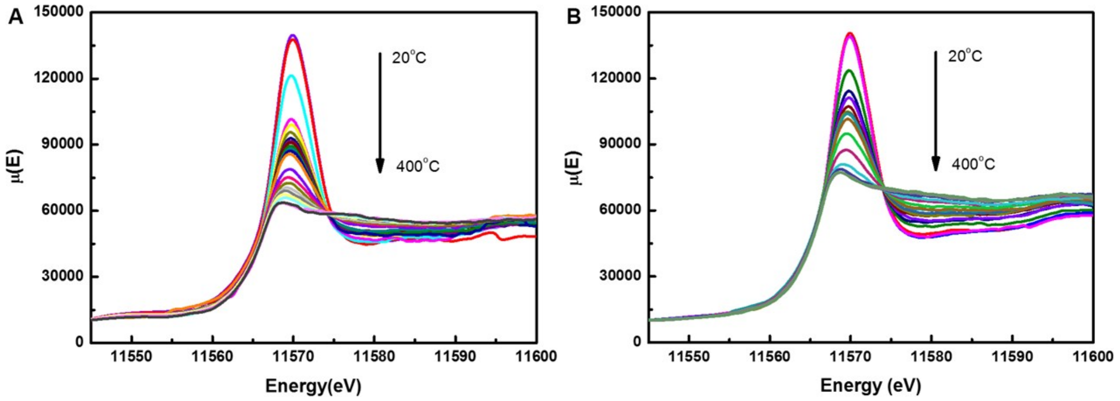


**Figure S4.** In-situ X-ray absorption near edge structure (XANES) spectra at Pt $L_{III}$-edge of (A) 2.16%Pt/FeOx and (B) 5.03%Na-2.16%Pt/FeOx catalysts during the reduction treatment process.

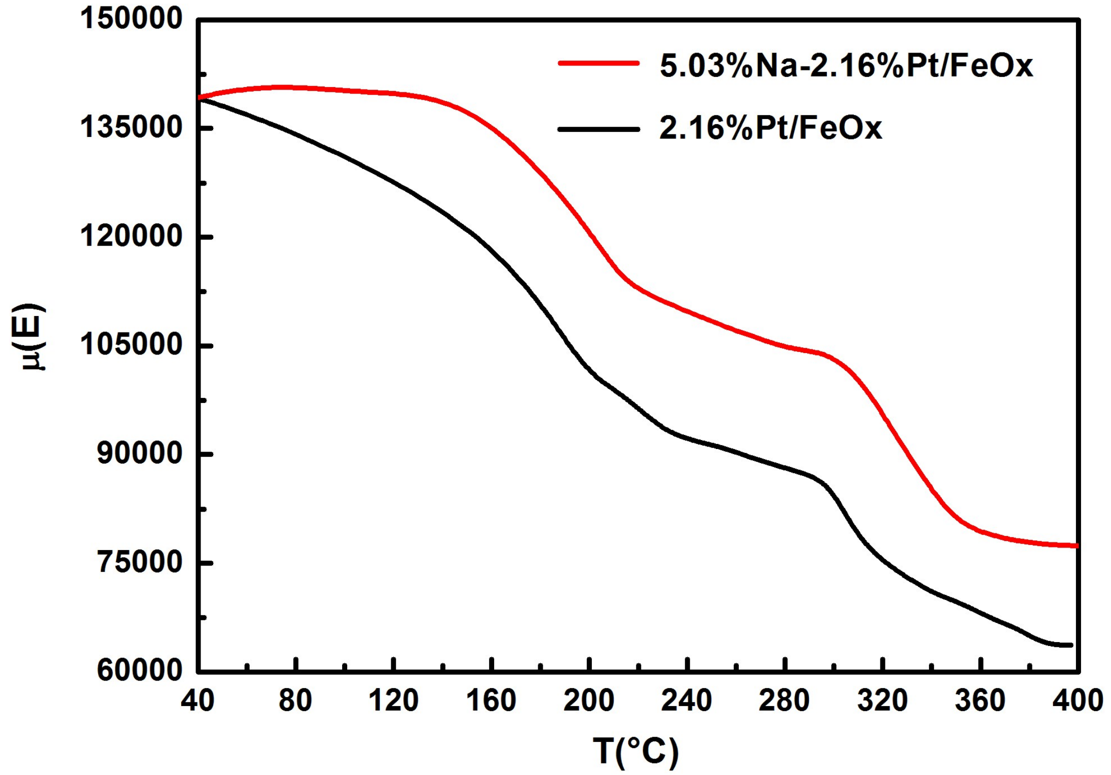


**Figure S5.** The whiteline intensity change of Pt with the reduction temperature.

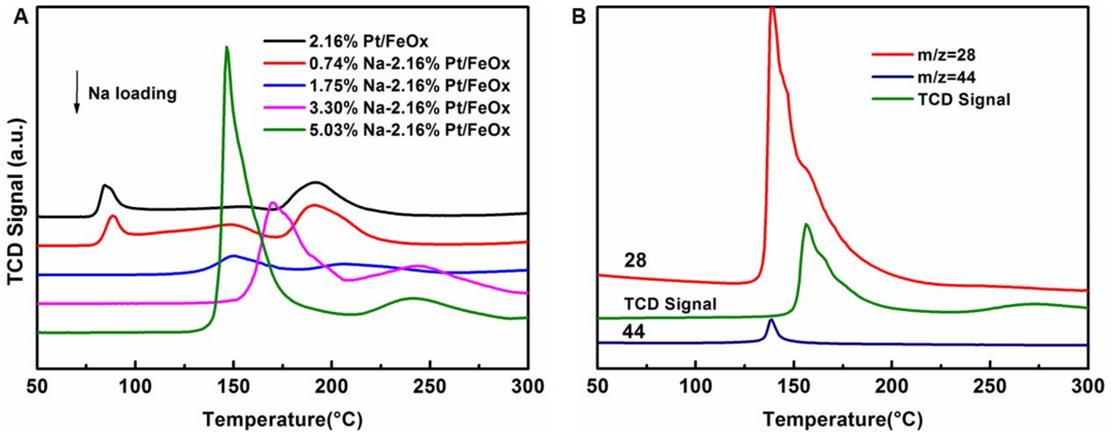


**Figure S6.** $H_2$-TPR profiles of catalysts with different Na loadings. The $H_2$ consumption peak at around 150 $^oC$ over the 5.03%Na-2.16%Pt/FeOx is due to the reductive decomposition of $NaNO_3$ (NO m/z = 28 and $N_2O$ m/z = 44).

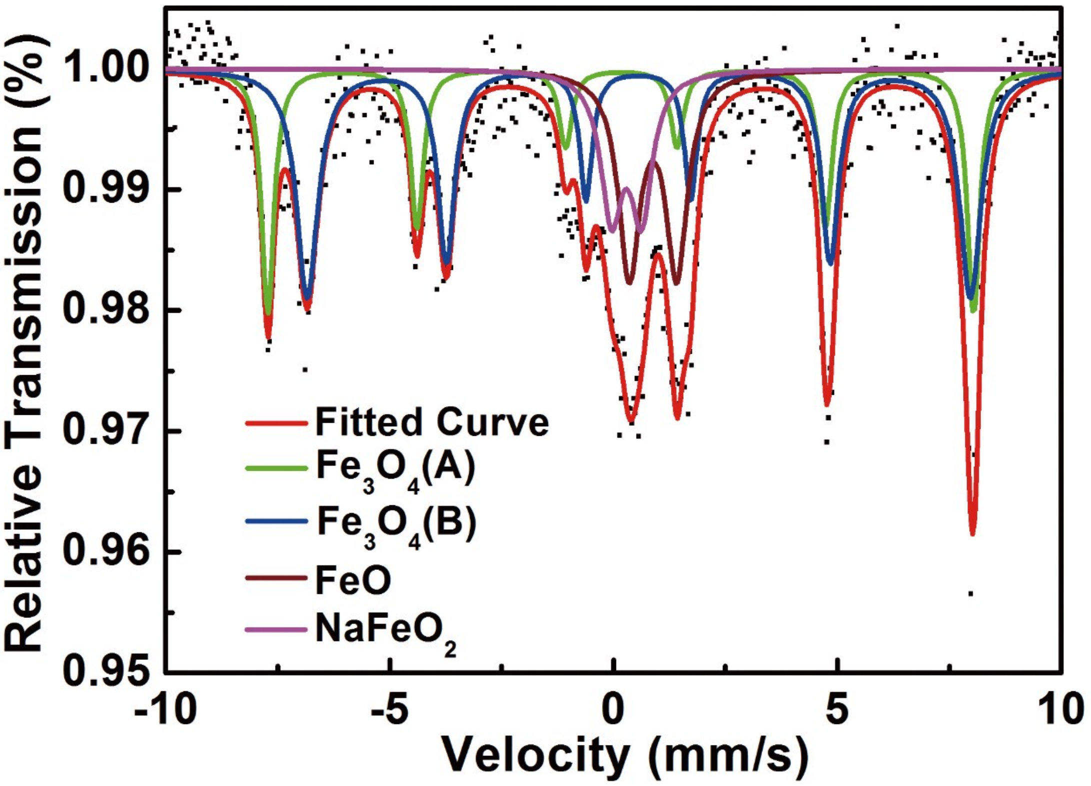


**Figure S7.** Mössbauer spectroscopy of $NaFeO_2$/FeOx support.

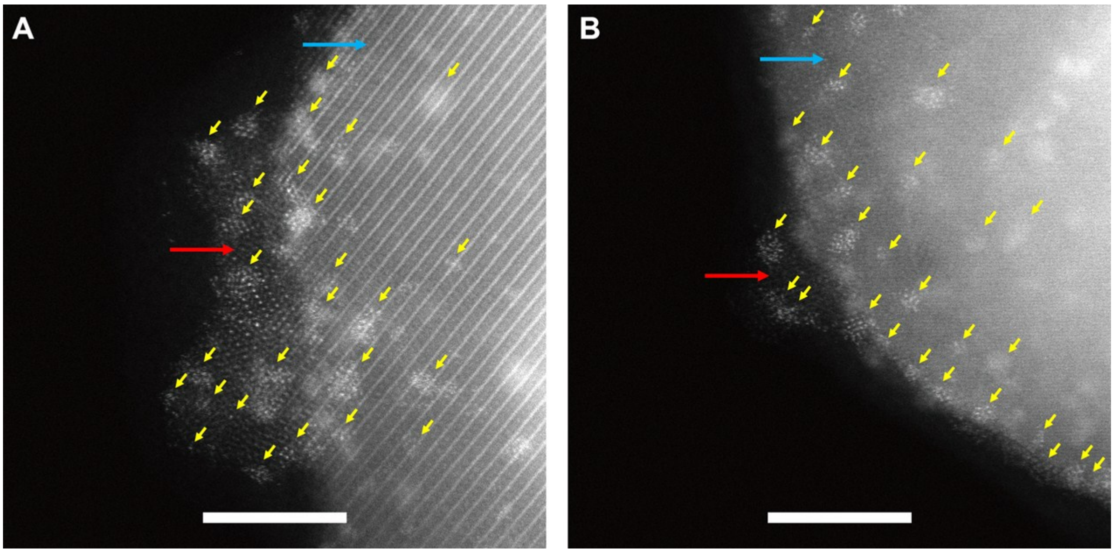


**Figure S8.** HAADF-STEM images of Pt/$NaFeO_2$/FeOx catalyst. Scale bar 5 nm. The yellow, red and blue arrows in the figures represent the assemblies of Pt single atoms, $NaFeO_2$ surface layer and $Fe_3O_4$ support, respectively.

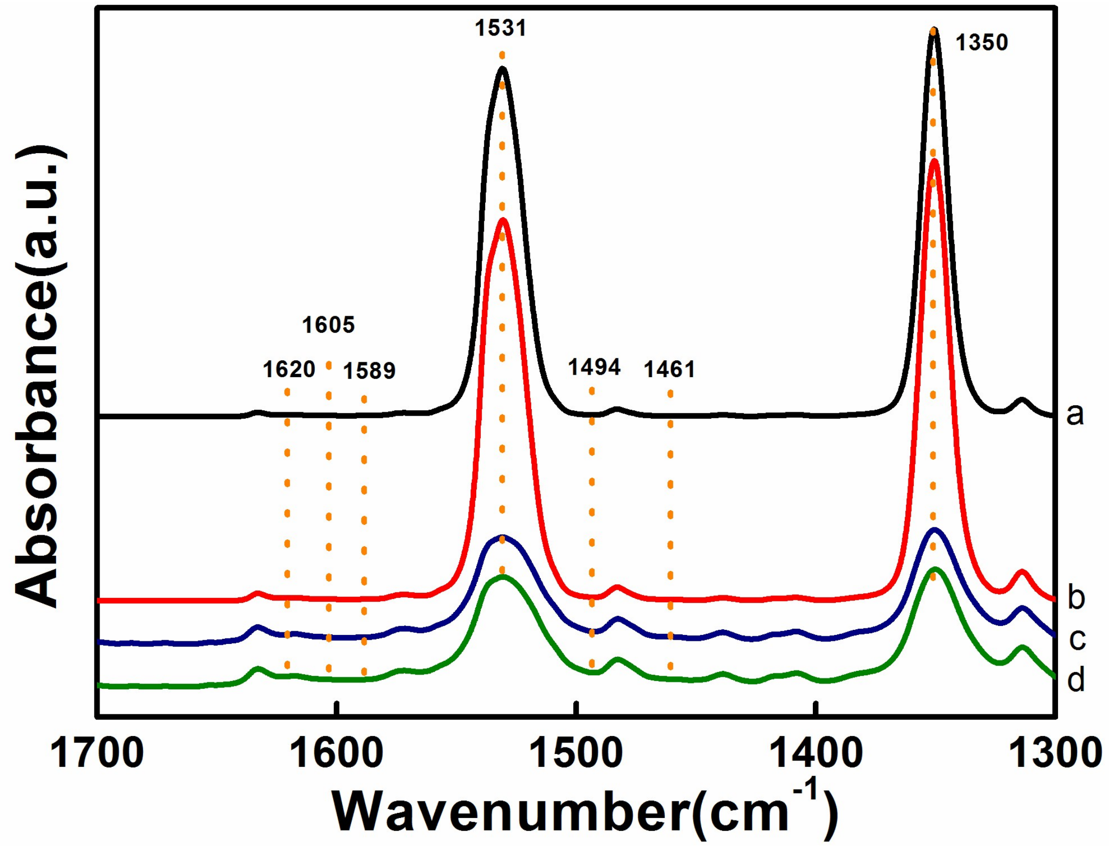


**Figure S9.** DRIFT spectra of 3-nitrostyrene adsorption at 80 °C on (a, b) 5.03%Na-FeOx-R400 and (c, d) FeOx-R400 before (a, c) and after (b, d) introduction of $H_2$.
